# Root Microbiome Assembly Underpins the Ecological Role of Emergent Macrophytes in a Pilot-Scale Constructed Wetland

**DOI:** 10.3390/biology15191739

**Published:** 2026-10-01

**Authors:** July Z. Florez, Lourdes Varela-Prieto, Ana Zárate, Aracelly Caselles-Osorio, Alejandro Llanos-Lizcano, Pablo Bruna

**Affiliations:** 1 Centro i~mar, MASH y CeBiB, Universidad de Los Lagos, Puerto Montt 5480000, Chile; 2 Laboratorio de Inmunología y Biología Molecular, Facultad de Química y Farmacia, Universidad del Atlántico, Barranquilla 080001, Colombia; lourdesvarela@mail.uniatlantico.edu.co; 3 Facultad de Ciencias de la Salud, Instituto de Ciencias Aplicadas, Universidad Autónoma de Chile, Temuco 4780000, Chile; ana.zarate@uautonoma.cl; 4 DVS Tecnología, Parcelación Neumann, Puerto Varas 5550000, Chile; 5 Centro de Estudios del Agua, Grupo de Investigación Humedales del Caribe Colombiano Facultad de Ciencias Básicas, Universidad del Atlántico, Barranquilla 080001, Colombia; aracaos9533@hotmail.com; 6 Department of Evolutionary Anthropology, University of Vienna, 1090 Vienna, Austria; alejandro.llanos.lizcano@univie.ac.at; 7 Human Evolution and Archaeological Sciences (HEAS), University of Vienna, 1090 Vienna, Austria; 8 Núcleo Científico y Tecnológico en Biorecursos (BIOREN), Universidad de La Frontera, Temuco 4780000, Chile; p.bruna01@ufromail.cl

**Keywords:** *Thalia geniculata*, *Cyperus articulatus*, microbial consortium, ecological network, assembly processes

## Abstract

Constructed wetlands (CWs) depend on plant–microbe associations for pollutant transformation, yet the processes linking macrophyte identity to rhizosphere microbiome assembly remain insufficiently resolved. We compared bacterial communities associated with *C. articulatus* (*Ca*) and *T. geniculata* (*Tg*) roots with an unplanted treatment (Up) in pilot-scale CWs on the Colombian Caribbean coast. Bacterial communities were characterized by 16S rRNA gene amplicon sequencing. Co-occurrence networks, taxon–environment correlations, and the phylogenetic bin-based null model iCAMP were used to assess community structure, potential associations and assembly mechanisms. *CaS* supported significantly greater bacterial diversity than *TgS* and Up, while each macrophyte recruited a distinct community. A shared core microbiome dominated by Rhodocyclaceae, Sphingomonadaceae, Chitinophagaceae, Pirellulaceae, and Xanthomonadaceae comprised taxa with putative roles in nutrient and organic matter transformations. Several bacterial families were significantly associated with PO_4_^3−^, NH_4_^+^, and dissolved oxygen. Assembly was predominantly stochastic, with dispersal limitation (45.2%) and ecological drift (33.2%) exceeding deterministic selection (16.5%). Nevertheless, planted treatments displayed higher network modularity and connectivity and inferred functional redundancy than Up, consistent with niche differentiation. These findings suggest that species-specific macrophyte configurations are associated with differences in root-associated microbial community composition, while assembly and network analyses provide exploratory insights into community organization. The limited replication precludes causal attribution to plant identity or conclusions regarding treatment performance. Future studies integrating controlled planting densities, increased replication, meta-omics, and root exudate characterization are needed to clarify plant–microbiome relationships and evaluate their potential relevance to constructed wetland functioning.

## Simple Summary

Emergent macrophytes influence constructed wetland performance through root microbiomes, yet host-associated microbial recruitment and community assembly processes remain poorly understood. Here, bacterial communities associated with *Cyperus articulatus* and *Thalia geniculata* roots were compared to those developing in unplanted wetland units. Each macrophyte species was associated with a distinct root microbiome, with *C. articulatus* exhibiting the greatest bacterial diversity, while both planted treatments displayed more complex exploratory microbial association networks than the unplanted units and shared a core group of bacterial families potentially involved in nitrogen, phosphorus, and carbon transformations. Community assembly analyses suggested greater contributions of dispersal limitation and ecological drift than deterministic selection, while macrophyte-specific planting configurations were associated with distinct root-associated microbial communities. Exploratory network and functional analyses further indicated potential differences in community organization and biogeochemical functions. These findings provide a basis for investigating plant–microbiome relationships and developing microbiome-informed constructed wetlands, although their implications for pollutant removal and operational stability require experimental validation.

## 1. Introduction

Constructed wetland (CW) ecosystems are multifaceted, highly efficient, and cost-effective ecotechnologies that integrate chemical, physical, and biological processes by employing aquatic plants (emergent, floating, or submerged macrophytes), substrates and associated microbial communities to remove nutrients and pollutants from wastewater, thereby contributing to environmental protection [1,2].

To enhance the pollutant removal efficiency of CWs, research has focused on several factors, particularly the selection of suitable macrophyte species [3,4]. More than 150 plant species have been reported worldwide in CWs for wastewater treatment [5], yet only a limited number are widely implemented in practice. The most commonly used are *Phragmites australis*, *Zizania latifolia*, *Typha angustifolia*, and *Typha latifolia* [6,7]. These emergent plants develop extensive root systems that generate a large rhizospheric surface, which supports the recruitment and colonization of specialized microbial groups [8,9,10]. Such plant–microbe interactions stimulate synergistic biogeochemical processes [11,12], ultimately enhancing the overall treatment performance of CWs [13].

It is well documented that the rhizospheric zone is a dynamic interface where physicochemical gradients and biological interactions converge, driving the recruitment, assembly, and functioning of root-associated microbiomes [14,15], thereby enhancing plant tolerance and strengthening defence mechanisms against pathogens [16,17].

Studies on *Phragmites australis*, *Typha latifolia*, *Thalia geniculata*, and other emergent wetland macrophytes consistently report an enrichment of Pseudomonadota (including Rhizobiales, Burkholderiales, and Pseudomonadales), Bacteroidota, Bacillota, and Actinobacteriota [18,19,20]. These taxa display wide metabolic versatility and plant growth-promoting attributes such as nitrogen fixation, phosphorus solubilization, and phytohormone production, pointing to reciprocal benefits between plants and their microbiota [8,21]. Together, they form a functional core root microbiome, many members of which contribute to nutrient mobilization and the detoxification of metals and organic contaminants, thereby sustaining key biogeochemical processes in CWs [22,23].

Conversely, only a limited number of studies have examined the root microbiomes of *Thalia geniculata* and *Cyperus articulatus* [24], highlighting the need for further research on microbial assembly that goes beyond simple assessments of richness and composition. Identifying the core root microbiome is crucial for understanding the effectiveness, stability, resilience, and functional capacity of these root-associated communities. Moreover, the interaction networks linking macrophytes with their microbial partners have been explored only marginally, constraining efforts to elucidate the overall stability and functioning of CW ecosystems [25,26].

Integrating ecological network analysis provides a powerful approach for exploring co-occurrence patterns, identifying keystone taxa, and inferring complex interactions within CW systems [19,27]. Previous studies have shown that higher microbial network complexity is associated with enhanced metabolic cooperation, which can, in turn, inform the optimisation of CWs design to improve performance, particularly with respect to contaminant removal [19,27,28]. Pilot-scale CWs provide an effective platform for investigating root microbe interactions because they replicate field conditions while allowing experimental control over key design parameters, including substrate composition and plant diversity.

In this study, we conducted pilot-scale CWs to compare the root-associated microbial communities of horizontal-flow CWs under unplanted (Up) conditions and those vegetated with two emergent macrophyte species using an integrative ecological framework combining high-throughput sequencing, community assembly analyses, and microbial network inference. Bacterial communities were characterized by Illumina MiSeq sequencing of the 16S rRNA gene, and the ecological mechanisms underlying community assembly were quantified using the phylogenetic bin-based null model iCAMP, allowing the relative contributions of deterministic processes (homogeneous and heterogeneous selection) and stochastic processes (dispersal limitation, homogenizing dispersal, and ecological drift) to be evaluated. Co-occurrence network analyses were further applied to identify keystone taxa and characterize patterns of microbial connectivity, modularity, and potential functional organization. We also investigated whether the core microbiome contained host-associated taxa occupying central topological positions within the ecological networks, suggesting disproportionate ecological importance.

Collectively, these analyses provide mechanistic insights into how emergent macrophytes regulate microbial community assembly and ecological interactions, establishing a scientific framework for engineering targeted plant–microbe partnerships and microbial consortia that enhance pollutant removal, improve the long-term sustainability of CWs, and advance biotechnological applications in phytodepuration [29,30].

## 2. Materials and Methods

### 2.1. Experimental Design, Sample Collection, and High-Throughput Sequencing

The setup, plant acclimatization, and transplant conditions of the pilot-scale horizontal subsurface flow constructed wetland (HSSF-CW) prototypes planted with *TgS* and *CaS* are described in Llanos-Lizcano et al. [24]. Briefly, the HSSF-CWs consisted of six concrete cells (0.94 m × 0.6 m × 0.4 m) arranged in parallel and installed at the wastewater treatment research facilities of Universidad del Atlántico, Barranquilla, Colombia (11°01′08″ N, 74°52′19″ W). Prior to the experiment, young specimens of *Ca* and *TgS*, previously reported as suitable for phytoremediation [1,31], were transplanted into the treatment units.

Two CW units were planted with 24 *TgS* individuals each, two units with 33 *CaS* individuals each, and the remaining two were maintained as Up controls. These planting densities corresponded to the species-specific configurations established for the pilot-scale HSSF-CWs [24] and were not intended as an experimental manipulation of plant density. Because *CaS* and *TgS* differ in plant architecture and growth characteristics, plant number cannot be considered equivalent to root biomass or rhizosphere extent. Consequently, comparisons between planted treatments represent the integrated effects of their respective vegetation configurations rather than effects attributable exclusively to plant identity.

Root samples (~1 g each) were randomly collected from the planted CWs (*TgS1*, *TgS2*, *CaS3*, and *CaS4*), whereas granular-medium samples (~1 g each) were obtained from the Up control CWs, resulting in a total of 23 initial samples. All samples were placed in sterile plastic bags, transported on ice (4 °C), and stored at −20 °C until DNA extraction.

The individual collections represented spatial subsamples rather than independent experimental replicates, because treatments were established at the CW level. To integrate within-unit spatial heterogeneity, material from the subsamples was pooled prior to DNA extraction, yielding five composite microbiome profiles: two *TgS*, two *CaS*, and one Up. Accordingly, the effective sample size for microbiome analyses was five composite profiles rather than 23 independent observations.

The pilot-scale design enabled the characterization of microbial responses under operationally relevant HSSF-CW conditions, providing a valuable basis for identifying treatment-associated patterns and generating mechanistic hypotheses. Nevertheless, the limited number of independent microbiome profiles, particularly the single Up composite, constrained statistical power and the estimation of within-treatment variability. Community comparisons were therefore interpreted primarily as exploratory, and the resulting patterns were considered preliminary evidence of treatment-associated microbial responses rather than definitive causal effects attributable to plant identity.

Genomic DNA was extracted from root and substrate samples using the PowerSoil™ DNA Isolation Kit (Qiagen, Hilden, Germany; previously MoBio) following the manufacturer’s protocol. DNA concentration and purity were measured using a NanoDrop One spectrophotometer (Thermo Scientific, Waltham, MA, USA), and DNA integrity was confirmed by 0.8% (*w*/*v*) agarose gel electrophoresis. Subsequently, 20 µL of pooled DNA was submitted to the Dalhousie University CGEB-IMR (https://www.dal.ca/study/program-sites/biochemistry-molecular-biology/research/resources---facilities.html, accessed on 18 February 2025) for 16S rRNA paired-end sequencing.

Environmental DNA from rhizosphere samples was amplified targeting the V5-V6 region of the 16S rRNA gene using specific primers (799F: 5′-AAC MGG ATT AGA TAC CCK G-3′; 1115R: 5′-AGG GTT GCG CTC GTT G-3′) [32]. Barcodes for sequencing multiplexing were added to the primers. The PCR mixture included HF buffer, dNTPs, BSA, primers, Phusion High-Fidelity DNA polymerase, and template DNA, with cycling conditions optimized for denaturation, annealing, and extension. The resulting libraries were sequenced using an Illumina MiSeq platform (Illumina, San Diego, CA, USA) (2 × 300 bp paired-end reads; 600-cycle v3 kit).

### 2.2. Data Processing and Statistical Analysis

Normalization and rarefaction of the microbiome data were performed using the MicrobiomeAnalyst platform (https://www.microbiomeanalyst.ca//ModuleView.xhtml, accessed on 18 February 2025) [33,34]. Raw sequences were analyzed with DADA2 [35], including quality filtering, dereplication, sample inference, chimera removal, and paired-end read merging. Following ASV inference, low-abundance and low-prevalence features were filtered using a minimum count of four reads and a 20% prevalence criterion, as specified in the MicrobiomeAnalyst documentation and implemented within the workflow described by Chong et al. [33]. These filters aimed to reduce sparsity and the influence of poorly represented features [36]. Low-variance features were subsequently removed based on the interquartile range. Filtering was analytical rather than an ecological definition of rarity, recognizing the potential importance of low-abundance taxa. Remaining features were normalized using total sum scaling (TSS) and taxonomically assigned against SILVA-SSU v138.1 [37].

Furthermore, the species richness, evenness, and microbial diversity indices (including Shannon and Simpson) were calculated. In addition, the treatments were compared by the non-parametric Mann–Whitney method with the *p*-value adjusted using the FDR method. Similarly, non-metric multidimensional scaling (nMDS) based on Bray–Curtis dissimilarities was used to visualize differences in species-level community composition, while heatmaps were generated using Ward’s hierarchical clustering method, with all analyses performed within the same platform.

The core microbiome analysis was conducted at different taxonomic levels, with a sample prevalence of 20% and a relative abundance of 0.01. Furthermore, we performed a pattern search to identify microbiome interactions across wetland treatments using Spearman rank correlation, with a correlation threshold of at least 0.3 and a *p*-value less than 0.05. The linear discriminant analysis (LDA) was used for the biomarker analysis.

The platform performs a non-parametric factorial Kruskal–Wallis sum-rank test to identify features with significant differences in abundance regarding the class of interest, followed by LDA to calculate the effect size of each differentially abundant feature. The features are considered significant depending on their adjusted *p*-value. The default adj. *p*-value cutoff is 0.05, and the LDA score is 3.0.

Influent and effluent water quality was monitored by determining chemical oxygen demand (COD), ammonium (NH_4_^+^), and orthophosphate (PO_4_^3−^) following Standard Methods. Water temperature, pH, oxidation–reduction potential (ORP), and dissolved oxygen (DO) were measured in situ using a multiparameter probe (WTW Multi 3420, WTW Company, Weilheim, Germany). Removal efficiencies for COD, NH_4_^+^, and PO_4_^3−^ were calculated from influent and effluent concentrations as described by Llanos-Lizcano et al. [24]. Physicochemical variables were integrated with bacterial community composition using redundancy analysis (RDA), implemented in the vegan package [38] in R software version 4.3.1 (R Core Team, Vienna, Austria), to explore associations between environmental gradients and community structure. Spearman rank correlations were additionally calculated between bacterial families and physicochemical variables and visualized using the corrplot package (R software version 4.3.1). The RDA and correlation patterns were interpreted as exploratory associations rather than evidence of direct environmental control or causal relationships.

### 2.3. Community Assembly Processes and Functional Prediction

Community assembly processes were explored using the normalised stochasticity ratio (NST) and the phylogenetic bin-based null-model framework iCAMP [39]. The taxonomic normalized stochasticity ratio (tNST) was calculated using Bray–Curtis dissimilarity, with values <0.5 and >0.5 indicating a predominance of deterministic and stochastic assembly, respectively. iCAMP was subsequently used to partition community turnover among ecological process categories within phylogenetic bins.

Because estimation of within-treatment community turnover requires at least two independent samples, the unplanted treatment (Up), represented by a single composite profile, was excluded from the iCAMP analysis. The analysis therefore included two independent *C. articulatus* profiles and two independent *T. geniculata* profiles. Following exclusion of Up, ASVs with zero abundance across all four remaining profiles were removed before matching the community table with the taxonomy table and phylogenetic tree. This procedure retained 2762 of the 3732 ASVs detected in the original dataset (74.0%) for subsequent iCAMP analysis.

Community turnover was partitioned with 1000 null-model randomizations. Abundance-weighted beta mean pairwise distance (bMPD) was used to quantify between-community phylogenetic turnover. Phylogenetic randomization was performed within bins, whereas taxonomic randomization was conducted across the complete retained taxon pool. Ecological processes were assigned using the Confidence index. The 1000 randomizations were used to generate null expectations for ecological-process classification, while the relative contribution of each process was estimated from abundance-weighted process assignments across phylogenetic bins. Because each macrophyte treatment comprised only two independent profiles, its within-treatment estimate was derived from a single pairwise community comparison. Accordingly, no inferential comparisons of process contributions between treatments were performed, and iCAMP estimates were interpreted descriptively.

Putative microbial functions were inferred using Functional Annotation of Prokaryotic Taxa (FAPROTAX) [40], which maps taxonomic assignments to literature-based ecological and metabolic functions, including nitrification, denitrification, and fermentation. FAPROTAX outputs were interpreted as predictions of functional potential rather than direct measurements of microbial metabolic activity. Differences in predicted functional profiles among CW configurations were explored using two-sided Welch *t*-tests implemented in STAMP [41].

### 2.4. Ecological Association Networks and Putative Central Taxa

Microbial association networks were explored to characterize patterns of bacterial ASV co-variation across the pilot-scale HSSF-CWs. Pairwise Spearman rank correlations were calculated among ASVs across the five composite microbiome profiles, integrating the two *Ca*, two *Tg* and one Up profiles into a single analysis. Given the limited number of independent observations, separate treatment-specific networks were not inferred. Correlations with |ρ| ≥ 0.3 and *p* < 0.05 were initially screened, and associations exceeding |ρ| > 0.75 with *p* < 0.01 were retained for network construction. These thresholds were used as exploratory selection criteria rather than as evidence of statistically validated ecological interactions.

Spearman correlations were calculated directly from ASV abundance data and therefore did not explicitly account for the compositional nature of amplicon-sequencing data. Accordingly, the resulting network was used to explore patterns of ASV co-variation within the integrated pilot-scale system, recognizing that sequencing depth and the number of detected ASVs do not substitute for independent biological replication. Although associations and network topology were calculated at the ASV level, taxonomic patterns were summarized and visualized at the family level to facilitate ecological interpretation of community structure. Thus, highly connected ASVs belonging to the same bacterial family were represented according to their family-level taxonomic assignment, while the underlying associations remained derived from ASV-level correlations.

Network topology was characterized using degree and betweenness centrality (BC), with BC quantifying the frequency with which a node occurs along the shortest paths connecting other nodes [42]. ASVs exhibiting relatively high centrality were considered putative central taxa rather than confirmed keystone taxa because their identification was based on network topology rather than direct measurements of metabolic activity, ecological function, or effects on other community members [43]. Network structure was explored and visualized using Gephi version 0.9.2 [44] with the Yifan Hu proportional force-directed layout (relative strength = 2) [45]. Network statistics and centrality patterns were interpreted descriptively as hypothesis-generating associations requiring validation with greater, independent replication.

## 3. Results

Five composite microbiome profiles from the pilot-scale HSSF-CWs were characterized, comprising two independent profiles associated with *CaS*, two associated with *TgS*, and one from the unplanted configuration (Up). Thus, the effective biological sample size for microbiome characterization was five composite profiles, with two independent experimental units represented for each planted configuration and one for Up.

A total of 286,159 raw sequencing reads were generated across the five profiles. Following initial quality filtering, 206,681 reads (72.2%) were retained, and subsequent denoising, paired-read merging, and chimera removal yielded 62,465 non-chimeric sequences (21.8% of the initial reads) for downstream microbial community characterization (Table S1). Rarefaction curves approached an asymptote for all five profiles, indicating that sequencing depth adequately represented the bacterial diversity recovered within the individual samples (Figure S1). This sequencing coverage supports the characterization of the observed communities but does not increase the number of independent biological replicates or overcome the limited replication available for treatment-level statistical inference.

### 3.1. Alpha-Diversity and Bacterial Structure Analysis Across Wetland Treatments

The primary features of microbial community variation were characterized using α-diversity metrics and taxonomic composition across the experimental configurations of the pilot-scale CWs. Alpha-diversity analysis showed higher Shannon and inverse Simpson diversity indices in CaS than in TgS and Up, whereas Chao1-estimated richness showed a different pattern, with the highest value observed in Up (Figure S2). Given the limited biological replication (*CaS*, *n* = 2; *TgS*, *n* = 2; Up, *n* = 1), these differences are reported as descriptive patterns rather than statistically confirmed treatment effects.

Regarding bacterial community structure, taxonomic abundance distributions were examined across the experimental configurations. Considering all samples, Pseudomonadota was the most abundant phylum (45.3%), followed by Bacteroidota (12.6%), Acidobacteriota (8.7%), Bacillota (8.6%), Chloroflexota (8.1%), Verrucomicrobiota (4.5%) and Planctomycetota (4.2%).

**Figure 1:**
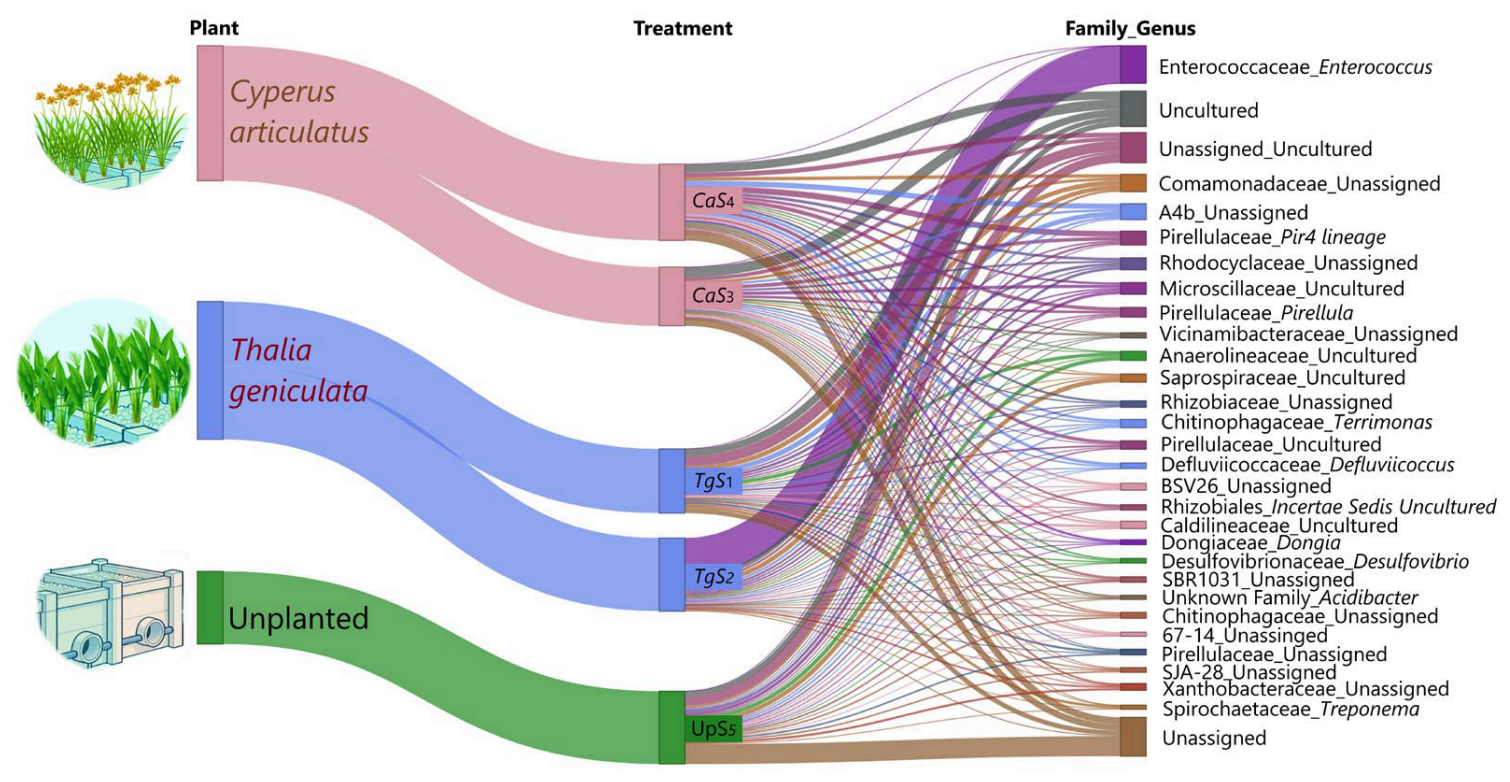
Distribution of dominant bacterial taxa across planted and unplanted CW treatments. Flow widths are proportional to taxon abundance, linking macrophyte species (*C. articulatus*, *T. geniculata*) and unplanted controls to individual treatments and their associated bacterial taxa, thereby highlighting host-specific microbial recruitment and taxonomic turnover across experimental conditions.

The distribution of dominant bacterial taxa across the CW configurations and their available replicates revealed both shared and configuration-associated patterns (Figure 1). The largest flows corresponded to a limited number of highly represented taxa, whereas the remaining bacterial community was distributed among numerous low-abundance taxa. Across the dataset, several bacterial lineages were consistently represented, including Comamonadaceae, Anaerolineaceae, Pirellulaceae, Rhodocyclaceae, Microscillaceae, Chitinophagaceae, and Rhizobiaceae, indicating a shared component of the CW microbiome. Their relative contributions nevertheless varied among *CaS*, *TgS*, and Up, resulting in different taxonomic profiles across the experimental configurations.

The two available profiles within each planted treatment showed broadly comparable taxonomic distributions, whereas differences in flow magnitude among configurations reflected shifts in the relative representation of bacterial taxa. The Up profile showed a comparatively greater contribution of unassigned taxa, whereas the vegetated configurations exhibited broader distributions across multiple classified bacterial taxa (Figure 1). These patterns are interpreted descriptively given the limited number of independent profiles.

**Figure 2:**
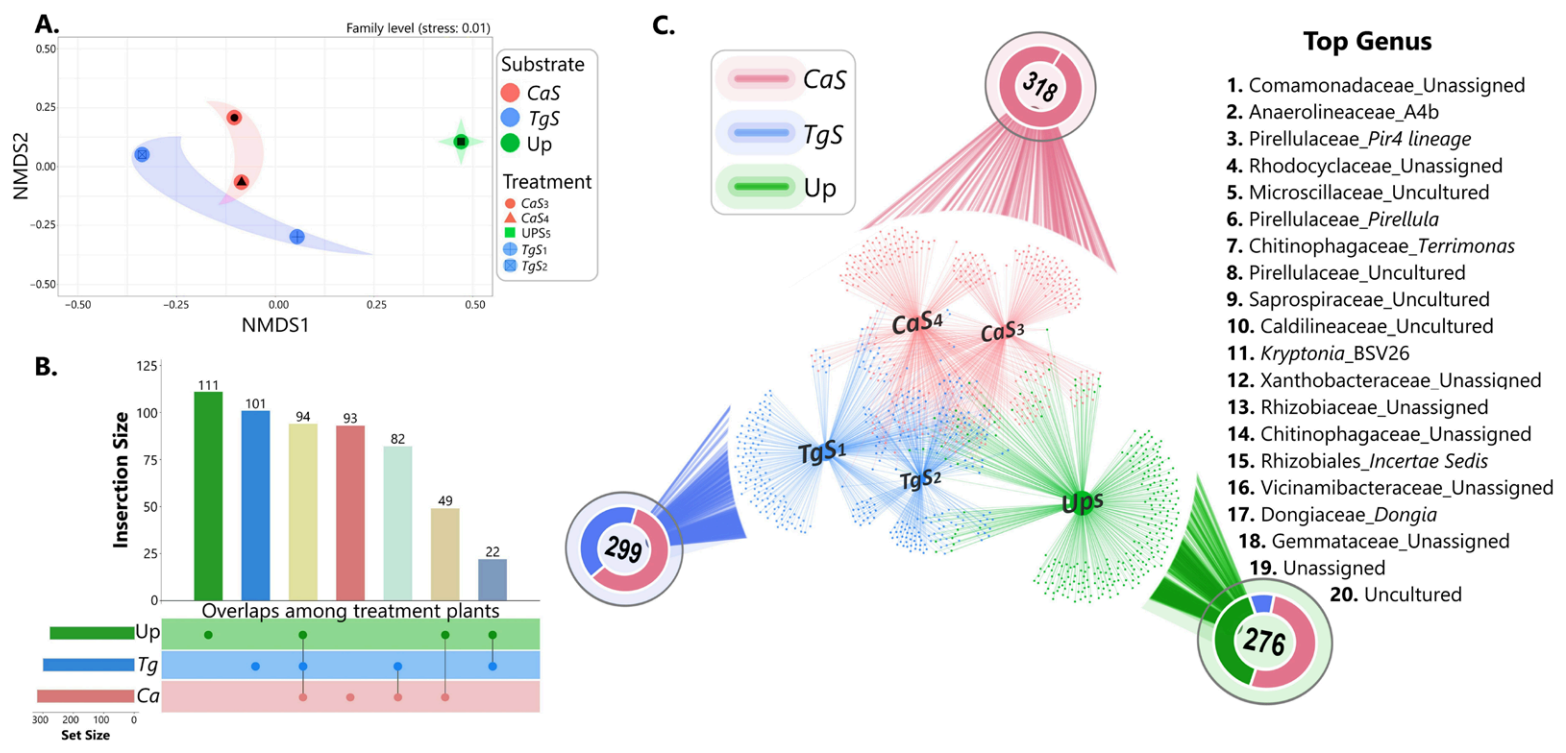
Taxonomic characteristics and linkages between experimental treatments. (**A**) NMDS ordination based on Bray–Curtis dissimilarity, illustrating compositional relationships among root-associated microbiomes of macrophytes in a CWs. (**B**) UpSet plot showing genus-level ASV presence/absence across treatments (*CaS*, pink; *TgS*, blue; Up, green), where dots indicate set membership, and vertical bars represent intersection sizes (**C**) Presence/absence network depicting both the number and membership of overlapping ASVs across treatments; nodes are colored according to treatment.

Beta-diversity analysis further indicated variation in bacterial community composition across the CW configurations, with the *CaS*, *TgS*, and Up profiles occupying different regions of the ordination space (Figure 2A). Presence–absence patterns likewise showed differences in ASV occurrence among configurations (Figure 2B,C). Given the limited replication, particularly the single Up profile, these patterns do not constitute confirmatory evidence of treatment-level separation.

The UpSet plot further illustrated differences in genus occurrence among the experimental configurations (Figure 2B). A substantial proportion of genera were detected exclusively within a single configuration, with 111 genera occurring only in Up, 101 only in *TgS*, and 93 only in *CaS*. In contrast, 94 genera were detected across all three configurations, representing the shared fraction of the bacterial community. Pairwise intersections were smaller, with 82 genera shared exclusively between *TgS* and *CaS*, 49 between Up and *CaS*, and 22 between Up and *TgS*.

The presence–absence network further highlighted differences in bacterial assemblages among treatments (Figure 2C), with taxa organized into treatment-associated subnetworks centered on *CaS*, *TgS*, and Up, while a subset of nodes connected multiple treatments and represented shared bacterial taxa; the *CaS* subnetwork contained the largest number of associated taxa (318), followed by *TgS* (299) and Up (276), and the most abundant genera across the network included members of Comamonadaceae, Anaerolineae, Pirellulaceae, Rhodocyclaceae, Microscillaceae, Chitinophagaceae, and Rhizobiaceae.

**Figure 3:**
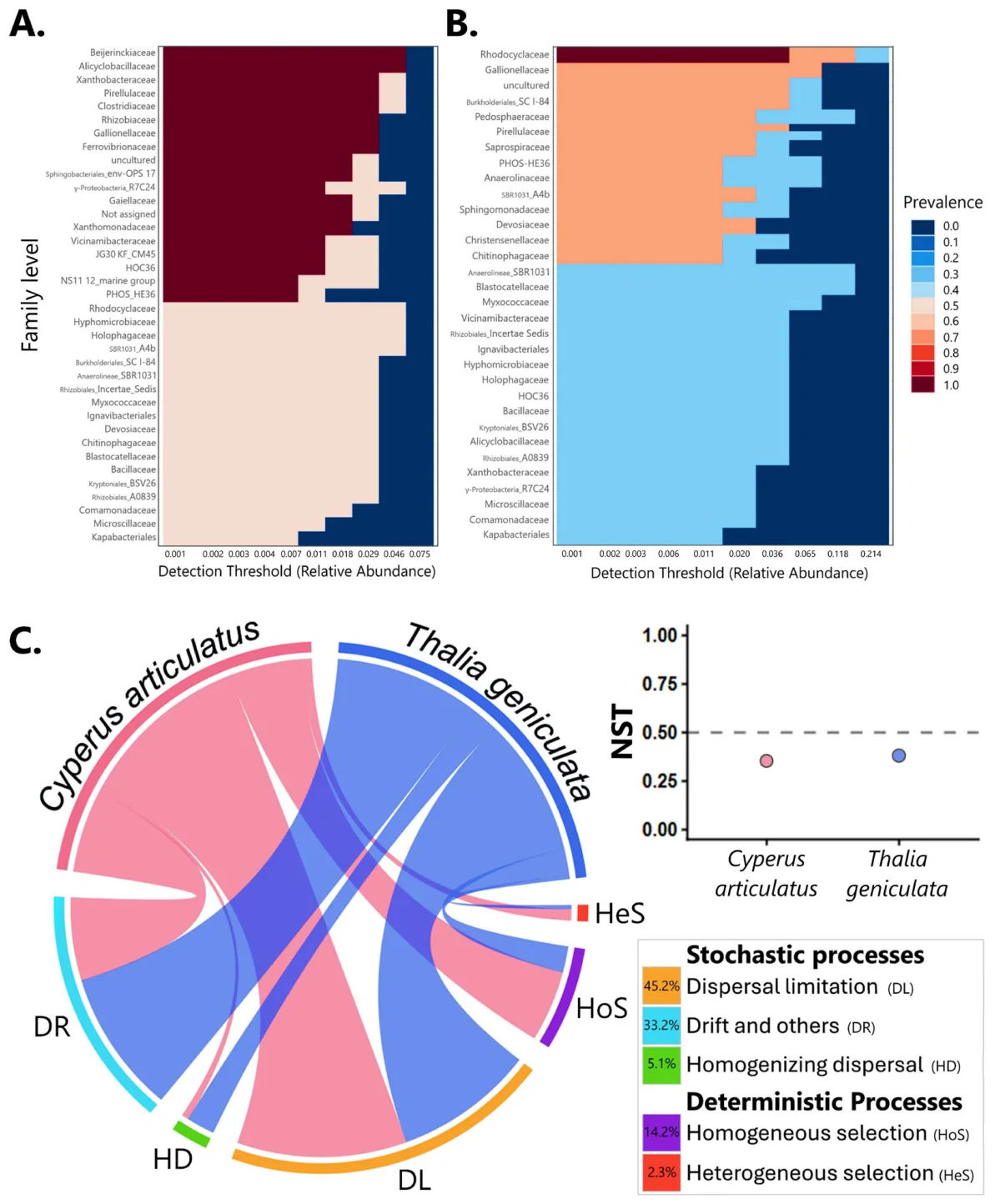
Heatmaps of the core microbiome showing the most prevalent bacterial taxa in (**A**) *C. articulatus* and (**B**) *T. geniculata* in wetland treatments. (**C**) Average relative contributions of ecological processes governing bacterial community assembly across different wetland treatments, based on iCAMP analysis.

Interestingly, at a prevalence threshold of 20% across CW treatments, 41 bacterial families were identified as part of the core microbiome (Figure 3). The distribution of these families differed between the two macrophyte hosts, and the number of families with a prevalence of 60% was higher in the *CaS* treatment than in the *TgS* treatment. Specifically, 19 families were predominantly associated with the *CaS* treatment (Figure 3A), whereas 14 families were enriched in the *TgS* wetland treatment (Figure 3B).

Of the 3732 ASVs in the original community table, 2762 (74.0%) were retained after restricting the iCAMP analysis to the two replicated planted treatments and were assigned to 108 phylogenetic bins. The 970 excluded ASVs (26.0%), which occurred exclusively in the unplanted samples, represented 20.9% of the total sequence abundance in the original dataset. These ASVs were primarily assigned to Pseudomonadota (20.7%), Bacteroidota (17.5%), Planctomycetota (12.3%), Chloroflexota (9.4%), Cyanobacteria (7.8%), Acidobacteriota (5.9%), and Verrucomicrobiota (5.3%). No additional ASVs were excluded during phylogenetic matching or binning.

The exploratory iCAMP analysis indicated a predominance of stochastic-process assignments in both planted treatments, although the relative contributions of individual assembly processes differed between macrophytes (Figure 3C). Within *CaS*, stochastic processes accounted for 75.9% of the inferred community turnover, including dispersal limitation (DL; 49.6%), drift and other undominated processes (DR; 24.3%), and homogenising dispersal (HD; 2.0%). Deterministic processes accounted for the remaining 24.1%, comprising homogeneous selection (HoS; 20.8%) and heterogeneous selection (HeS; 3.3%). In *TgS*, stochastic processes accounted for 91.1% of the inferred turnover, with DR (42.0%), DL (40.8%), and HD (8.3%) contributing the largest proportions, whereas HoS and HeS accounted for 7.7% and 1.2%, respectively.

Across the two planted treatments, the arithmetic mean of the process contributions was 83.5% for stochastic processes (DL, 45.2%; DR, 33.2%; HD, 5.1%) and 16.5% for deterministic processes (HoS, 14.2%; HeS, 2.3%). These mean values summarize the overall pattern depicted in the chord diagram (Figure 3) and should not be interpreted as an independently replicated whole-system estimate. Because each treatment was represented by only two independent composite samples, the process contributions for each macrophyte were derived from a single within-treatment pairwise community comparison. Accordingly, no inferential statistical comparison of assembly process contributions between macrophyte treatments was performed.

**Figure 4:**
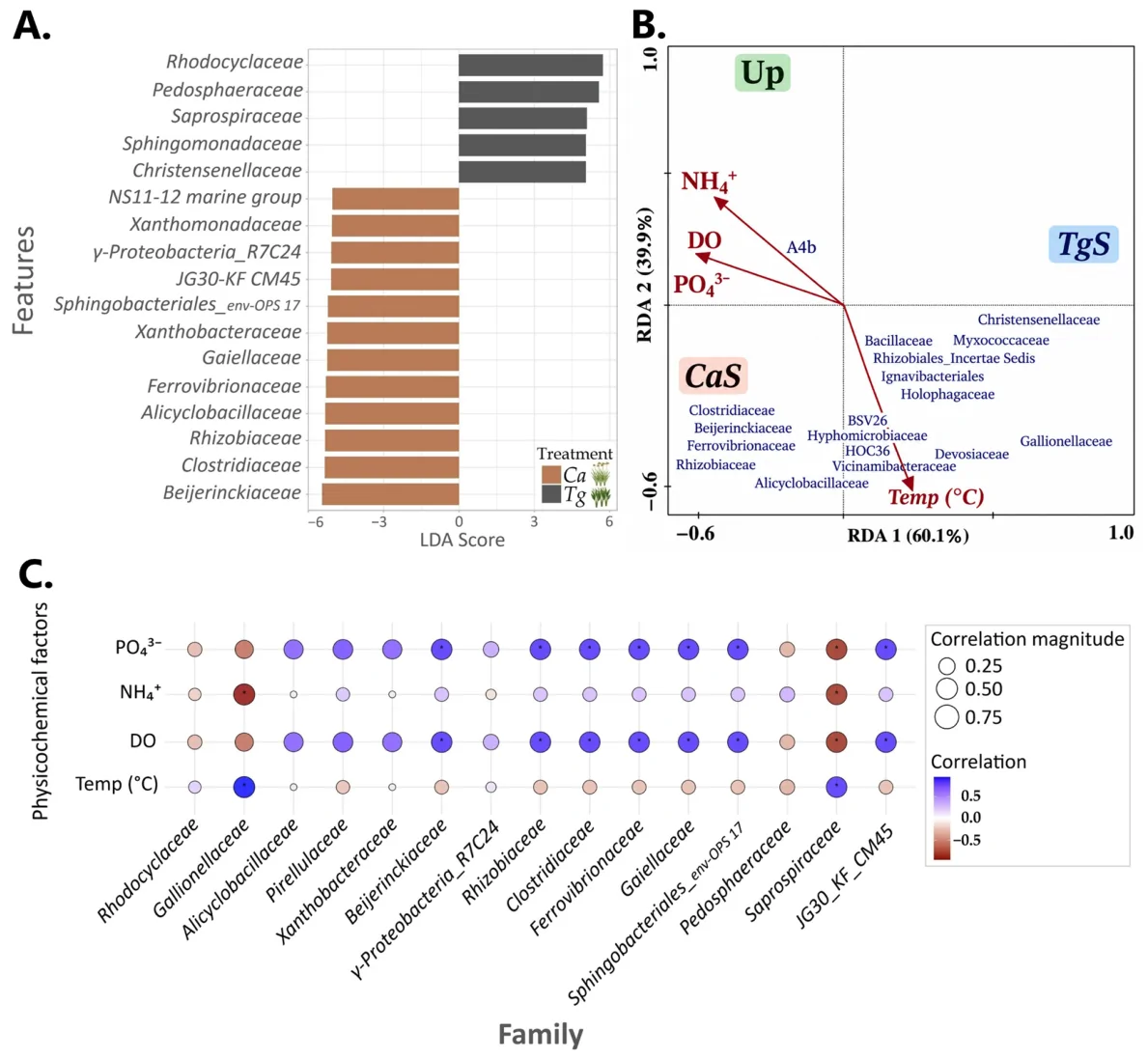
Variation patterns in the root-associated microbiomes of macrophytes in a pilot-scale CWs. (**A**) Linear discriminant analysis (LDA) and Effect Size analysis identify bacterial taxa at the family level that contribute significant differences to experimental treatments (*CaS* vs. *TgS*). LDA scores were calculated, and only bacterial taxa that raised a logarithmic score ≥ 3.0 are shown. (**B**) Redundancy analysis (RDA) including only the most relevant variables, selected through stepwise modeling with permutation tests. (**C**) Spearman correlation heatmap showing relationships between microbial communities and environmental variables, where blue denotes positive correlation coefficients and red denotes negative ones (* *p* ≤ 0.05).

Linear Discriminant Analysis (LDA) revealed twelve taxa contributing to the differentiation of the *CaS* profiles, including members of Alicyclobacillaceae, Beijerinckiaceae, Clostridiaceae, Ferrovibrionaceae, Gaiellaceae, Rhizobiaceae, Xanthobacteraceae, and Xanthomonadaceae. In *TgS*, five taxa contributed to the observed differentiation, including Christensenellaceae, Pedosphaeraceae, Saprospiraceae, Sphingomonadaceae, and Rhodocyclaceae (Figure 4A). Importantly, these taxa represent exploratory discriminant features rather than statistically validated treatment biomarkers.

Associations between bacterial community composition and physicochemical conditions (Table S2) were further explored using RDA (Figure 4B). The first two axes accounted for 60.1% (RDA1) and 39.9% (RDA2) of the constrained variation, respectively. Along RDA1, *CaS* profiles were positioned in the direction of the NH_4_^+^, PO_4_^3−^ and DO gradients. Clostridiaceae, Beijerinckiaceae, Ferrovibrionaceae, Rhizobiaceae, and Alicyclobacillaceae showed similar ordination patterns, whereas Myxococcaceae, Gallionellaceae, and Christensenellaceae were oriented toward the temperature gradient.

Consistent with the RDA, exploratory Spearman correlations showed negative relationships of Saprospiraceae with PO_4_^3−^, DO, and NH_4_^+^, whereas Gallionellaceae was negatively correlated with NH_4_^+^ and positively correlated with temperature. Conversely, Beijerinckiaceae, Rhizobiaceae, Ferrovibrionaceae, and Gaiellaceae showed positive correlations with DO and PO_4_^3−^ (Figure 4C).

Despite the limited biological replication, these multivariate and correlation patterns provide ecologically informative evidence of community–environment relationships under operationally relevant pilot-scale conditions, thereby identifying patterns and hypotheses that may not emerge under simplified experimental settings. Nevertheless, these patterns are interpreted as exploratory co-variation rather than evidence of direct environmental control or causal treatment effects.

### 3.2. The Interconnected Bacterial Assembly Across the Pilot-Scale CWs

**Figure 5:**
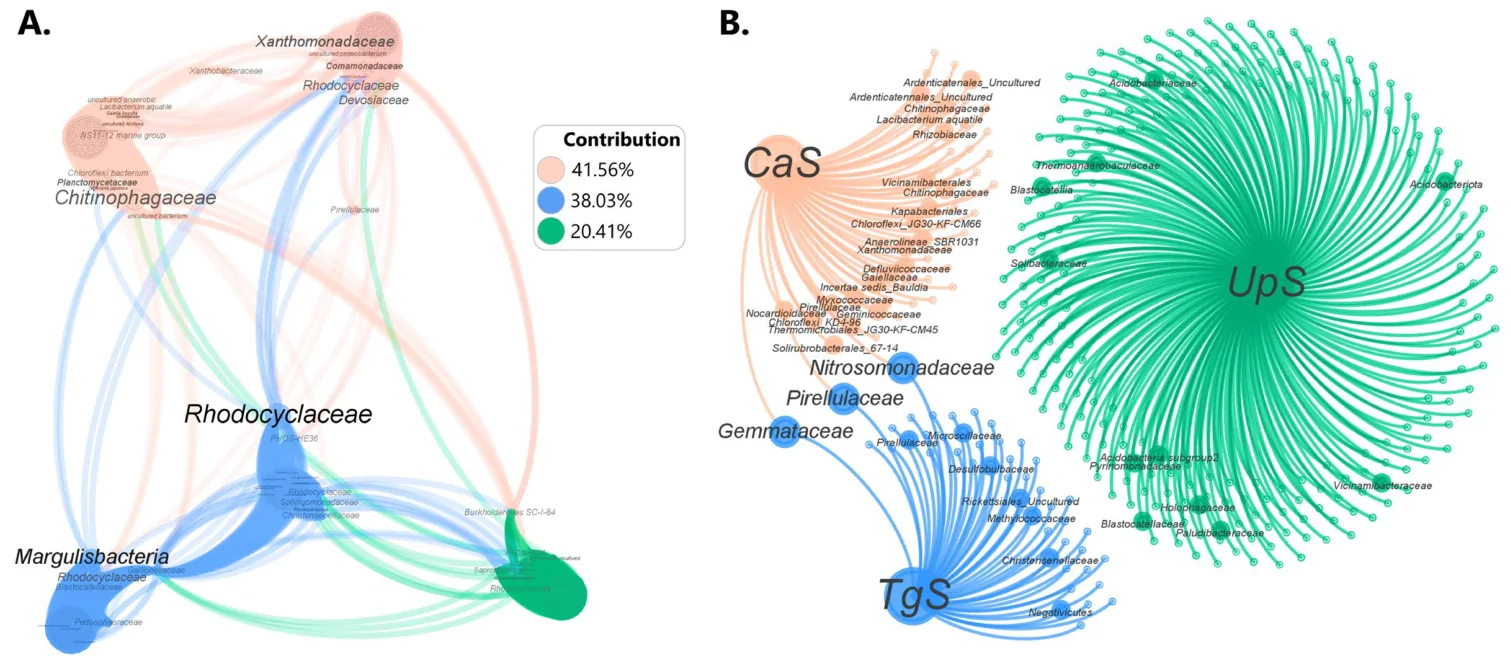
Based on Spearman rank correlations at the family level in root-associated macrophytes, significant co-occurrence patterns were observed among bacterial taxa across different experimental treatments in pilot-scale CWs. Network topology was characterized using betweenness centrality (**A**) and degree (**B**). Each node represents a bacterial ASV and is color-coded according to treatment (*CaS*: pink; *TgS*: blue; Up: green).

Ecological co-occurrence network analysis identified putative central taxa potentially involved in niche partitioning and microbial associations. Central taxa were identified based on betweenness centrality (i.e., nodes acting as bridges along shortest paths) and node degree (i.e., the number of direct correlations associated with each node), reflecting their relative topological importance within the network (Figure 5 and Table S3).

The exploratory correlation network comprised 1219 nodes and 157,681 edges (Table S3), with a modularity of 0.756, an average clustering coefficient of 0.498, an average path length of 1.608, and an average degree of 1.77. *CaS* and *TgS* were associated with a higher number of nodes and edges than Up; however, because the network was inferred jointly across the five composite profiles, these patterns describe the distribution of ASV associations within the dataset and were not interpreted as statistically supported differences in network complexity between treatments.

In *CaS*, central families included Alicyclobacillaceae, Chitinophagaceae, Devosiaceae, Nitrospiraceae, Pirellulaceae, Rhodocyclaceae, Sphingomonadaceae, Xanthobacteraceae, and Xanthomonadaceae. In contrast, *TgS* was associated with central taxa including Blastocatellaceae, Christensenellaceae, Leptospiraceae, Margulisbacteria, Pedosphaeraceae, Rhodobacteraceae, Rhodocyclaceae, and Sphingomonadaceae. Up was associated with Anaerolineaceae, Bacillaceae, PHOS-HE36, and Rhodocyclaceae as central taxa. Notably, Rhodocyclaceae showed high topological centrality across multiple experimental configurations, while several uncultured taxa occurred in association with both *CaS* and *TgS* (Figure 5A).

Furthermore, degree-based analysis identified Nitrosomonadaceae, Pirellulaceae, and Gemmataceae among prominent network taxa (Figure 5B).

**Figure 6:**
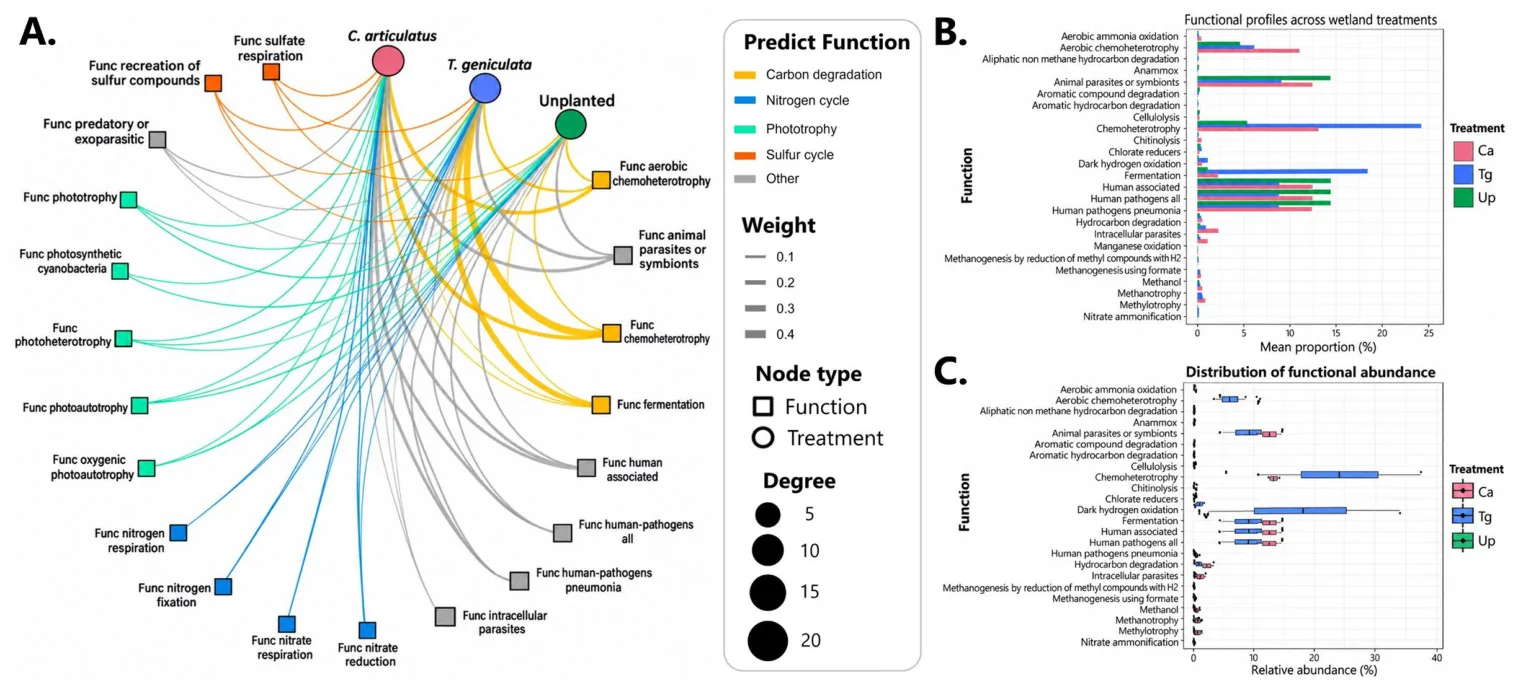
Predicted microbial functions across pilot-scale CW treatments. (**A**) Spider-plot linking treatments (*Cas*, *TgS*, Up) to dominant FAPROTAX functions; node size indicates degree, edge width indicates association weight, and colors denote functional categories. (**B**,**C**) Mean relative abundance (%) of selected functions among treatments.

FAPROTAX-based functional inference indicated that microbial communities across all wetland treatments were dominated by heterotrophic carbon-processing pathways, with chemoheterotrophy and fermentation consistently representing the most abundant predicted functions (Figure 6). However, the relative representation of these pathways varied among treatments. *TgS* showed the highest relative representation of chemoheterotrophy and fermentation, consistent with a predicted functional profile associated with organic matter decomposition, whereas *CaS* displayed a comparatively greater representation of aerobic chemoheterotrophy (Figure 6A).

The Up treatment showed a distinct predicted functional profile, with a higher relative representation of categories related to human pathogens and animal parasites or symbionts than the planted treatments. In contrast, nitrogen-, sulfur-, and methane-related pathways remained consistently rare across all treatments and represented only a minor fraction of the predicted functional repertoire. Specifically, functions associated with aerobic ammonia oxidation, nitrate reduction and respiration, nitrogen fixation, nitrate ammonification, methanotrophy, methylotrophy, methanol utilization, methanogenesis, and sulfur compound respiration occurred at very low relative abundances (Figure 6B,C). These relative-abundance patterns characterize the functional profiles observed in the analyzed samples and are not intended as inferential evidence of treatment-level functional differentiation.

Overall, planted treatments (i.e., *CaS* and *TgS*) exhibited more extensive and densely connected microbial association patterns spanning multiple putative functional groups than the Up treatment, suggesting potential differences in functional organization and ecological complexity that should be interpreted as exploratory rather than statistically validated treatment effects.

## 4. Discussion

Elucidating root microbiome interactions of emergent macrophytes provides critical opportunities to design plant–microbe partnerships that enhance pollutant removal [9,46] and support the long-term sustainability of CWs. In this study, we examined the root-associated microbiota of macrophytes in pilot-scale CWs located on a university campus on the Colombian Caribbean coast, as part of ongoing efforts to enhance the operational effectiveness of wastewater treatment systems.

The distinct microbial profiles associated with the macrophyte-specific planting configurations indicate that differences in plant-associated habitats are reflected in community organization under operationally relevant conditions, extending previous observations from constructed wetlands by linking taxonomic composition with complementary ecological descriptors within the same experimental system [8,14,47].

The observed compositional differences are consistent with the capacity of macrophytes to generate heterogeneous rhizosphere environments through root architecture, organic compound exudation, and radial oxygen loss [48,49,50]. These processes can influence substrate availability, redox conditions, and microbial niche differentiation, potentially contributing to the recruitment of distinct bacterial assemblages [18,51,52]. However, root exudation, oxygen release, and associated microscale gradients were not directly measured in the present study. Therefore, their potential contribution to the observed treatment-associated patterns remains a plausible ecological explanation rather than a demonstrated mechanism.

The assembly analyses add a further dimension to this interpretation, with iCAMP attributing the largest relative contribution to dispersal limitation and with tNST values remaining below 0.5. Rather than indicating a single dominant mechanism, these findings illustrate how different null-model frameworks capture complementary aspects of community assembly, consistent with ecological theory describing the interacting influences of dispersal, selection, and drift on microbial community composition [53,54,55]. Nevertheless, limited replication and the phylogenetic coverage of iCAMP constrain the interpretation of these estimates, which cannot independently establish restricted dispersal or plant-mediated selection.

The taxonomic profiles revealed differences in core community membership and the relative representation of bacterial families between *CaS* and *TgS*, consistent with previous evidence that plant-associated microbiomes vary across host and environmental conditions [54,56,57]. The greater representation of certain families in *CaS* therefore indicates a distinct root-associated community composition under its established planting configuration, although these differences do not establish greater temporal stability, functional redundancy, or resilience, which would require direct assessment of community persistence and functional responses.

The network analysis extends the compositional findings by revealing differences in the inferred patterns of microbial association between the root-associated communities of *CaS* and *TgS*, suggesting that macrophyte-specific planting configurations are associated not only with taxonomic composition but also with community organization. This interpretation is consistent with studies reporting variation in root-associated microbiomes across plant species, root compartments, and local environmental conditions [4,52,58]. However, given the limited replication and exploratory nature of the analysis, these patterns should be interpreted as hypotheses regarding microbial community organization rather than evidence of differences in ecological interactions, network complexity, or underlying assembly mechanisms.

The taxonomic identity of central families provides additional ecological context, as Nitrospiraceae and Xanthobacteraceae contain members involved in nitrogen transformations, whereas Rhodocyclaceae and Chitinophagaceae include taxa associated with nitrogen cycling and organic matter degradation, respectively [59,60,61]. Their occurrence and centrality identify microbial groups of potential functional interest.

In this context, taxonomy-based assessment of the potential functional repertoire of the root-associated microbial communities [40], revealing a predominance of chemoheterotrophic and other organic-carbon-associated categories consistent with the importance of heterotrophic microorganisms in wastewater treatment systems. Within this overall pattern, *TgS* showed greater predicted representation of fermentation and chemoheterotrophy, whereas *CaS* showed greater representation of aerobic chemoheterotrophy and animal parasite- or symbiont-associated categories, indicating that differences in community composition were accompanied by differences in the functional annotations of their constituent lineages.

Nitrogen-related functions, including nitrate reduction and nitrogen fixation, were also predicted at comparatively low relative abundances, together with categories associated with sulfur transformations, phototrophy, and methanogenesis. These annotations suggest that the root-associated communities contain lineages with diverse metabolic potential, although taxonomy-based predictions do not establish functional gene abundance, gene expression, metabolic activity, or process rates and therefore require validation through direct functional measurements.

Furthermore, the unplanted configuration provides a reference for interpreting the plant-associated microbiomes, as its distinct taxonomic and network profiles, including the prominence of Anaerolineaceae and Bacillaceae, contrast with the broader distribution of bacterial families observed in the planted units. These differences are consistent with the capacity of macrophyte roots to modify local physicochemical conditions through oxygen transport and organic substrate release [60,62,63], although these processes were not directly quantified in the present study and their contribution to microbial community organization remains to be determined.

Previous work on the same pilot-scale units reported higher NH_4_^+^ and PO_4_^3−^ removal efficiencies in planted configurations [24,64], providing an operational context for the present microbiome findings without establishing a causal relationship between microbial composition, inferred network structure and treatment performance. The occurrence of taxa associated with biogeochemical transformations therefore identifies candidates for subsequent investigation rather than demonstrating that the observed microbial differences explain the previously reported removal efficiencies.

Together, these findings demonstrate an association between macrophyte-specific planting configuration and root-associated microbial community composition, accompanied by differences in inferred network organization and taxonomy-based functional annotations. Because plant identity was not independently separated from planting density and other configuration-related factors, and because the single Up composite and different sampling matrices constrain comparisons with the unplanted control, the results should be interpreted as exploratory evidence supporting future studies with balanced replication, standardized sampling, direct functional measurements, and explicit assessment of root-associated environmental conditions.

## 5. Conclusions

From an applied perspective, this pilot-scale study provides evidence that macrophyte-specific planting configurations are associated with distinct root-associated microbial communities in horizontal subsurface flow CWs. Differences in root architecture, oxygen release, and exudation may contribute to these patterns, although their individual effects were not directly quantified.

The planted systems harbored taxa associated with nitrogen, phosphorus, and carbon transformations, including organic matter degradation, while taxonomy-based functional annotations and exploratory assembly and network analyses suggested differences in potential microbial functions and community organization. These findings should be interpreted within the constraints of the experimental design, as plant identity was not independently disentangled from planting density and other configuration-related factors, while the single Up composite profile and differences in sampling matrices further limit comparisons with the unplanted control.

Accordingly, future research should incorporate balanced replication, standardized sampling, direct measurements of pollutant removal and biogeochemical process rates, and characterization of root biomass, exudates, and rhizosphere environmental conditions. Complementary meta-omics and cultivation-based approaches could further clarify the ecological roles of uncultured and poorly characterized taxa. Establishing quantitative relationships among vegetation characteristics, microbial activity, and treatment performance will be essential to determine whether targeted plant–microbiome management can improve pollutant removal and operational stability, thereby supporting the development of microbiome-informed constructed wetlands for sustainable wastewater treatment.

## Data Availability

Raw 16S rRNA gene sequencing data generated in this study are available in the NCBI Sequence Read Archive (SRA) under BioProject accession PRJNA1285480. Processed ASV abundance tables, sample metadata, analysis scripts, and input and output files used for iCAMP and ecological association network analyses are publicly available through the study’s GitHub repository (https://github.com/pabruna/wetland-community-assembly, accessed on 8 September 2026). Physicochemical data associated with the experimental treatments and used in the environmental analyses are provided in Table S2 of the Supplementary Materials.

## Supplementary Materials

The following supporting information can be downloaded at https://www.mdpi.com/article/10.3390/biology15191739/s1, Figure S1. Rarefaction curve showing the bacterial species richness observed from different experimental treatments in a pilot wetland plant. Figure S2. Diversity indices at the family level in the root microbiomes of macrophytes (*C. articulatus* and *T. geniculata*) across different experimental treatments in a pilot wetland study (*n* = 5). The indices include A. Shannon, B. Inverse Simpson, C. Chao1, offering comprehensive insights into the microbial community structure. Table S1. Summary of raw sequencing reads and the number of reads retained at each quality trimming/filtering step (pre-processing) and denoising step (DADA2) for each experimental treatment (*TgS*, *CaS*, and Up) in the pilot-scale CWs. The final column shows the count of non-chimeric reads used for downstream analyses. Table S2. Mean concentration values ± standard deviation of physicochemical parameters for each experimental treatment (*TgS*, *CaS*, and Up) and influent wastewater. Table S3. Topological properties of co-occurrence networks in the root microbiomes of macrophytes from a pilot-scale CW.
