# Supplementary material for: Root Microbiome Assembly Underpins the Ecological Role of Emergent Macrophytes in a Pilot-Scale Constructed Wetland
Source: Biology (Basel). 2026 Oct 1;15(19):1739. doi: 10.3390/biology15191739 (PMC13663393; doi:10.3390/biology15191739)
Supplement: Supplementary file 1 [file biology-15-01739-s001.zip › biology-4537434-supplementary.pdf]

biology

MDPI

Supplementary Material

Root Microbiome Assembly Underpins the Ecological Role of Emergent Macrophytes in a Pilot-Scale Constructed Wetland

July Z. Florez [1,*,†], Lourdes Varela-Prieto [2], Ana Zárate [3,4,†], Aracelly Caselles-Osorio [5], Alejandro Llanos-Lizcano [6,7] and Pablo Bruna [8]

[1] Centro i~mar, MASH y CeBiB, Universidad de Los Lagos, Puerto Montt 5480000, Chile
[2] Laboratorio de Inmunología y Biología Molecular, Facultad de Química y Farmacia, Universidad del Atlántico, Barranquilla 080001, Colombia; lourdesvarela@mail.uniatlantico.edu.co
[3] Facultad de Ciencias de la Salud, Instituto de Ciencias Aplicadas, Universidad Autónoma de Chile, Temuco 4780000, Chile; ana.zarate@uautonoma.cl
[4] DVS Tecnología, Parcelación Neumann, Puerto Varas 5550000, Chile
[5] Centro de Estudios del Agua, Grupo de Investigación Humedales del Caribe Colombiano Facultad de Ciencias Básicas, Universidad del Atlántico, Barranquilla 080001, Colombia; aracaos9533@hotmail.com
[6] Department of Evolutionary Anthropology, University of Vienna, 1090 Vienna, Austria; alejandro.llanos.lizcano@univie.ac.at
[7] Human Evolution and Archaeological Sciences (HEAS), University of Vienna, 1090 Vienna, Austria
[8] Núcleo Científico y Tecnológico en Biorecursos (BIOREN), Universidad de La Frontera, Temuco 4780000, Chile; p.bruna01@ufromail.cl
* Correspondence: july.florezguerrero@ulagos.cl
† These authors contributed equally to this work.

Journal: biology

biology

Table S1

Summary of raw sequencing reads, and the number of reads retained at each quality trimming/filtering step (pre-processing) and denoising step (DADA2) for each experimental treatment (*Tg, Ca*, and Unplanted) in the pilot-scale constructed wetland. The final column shows the count of non-chimeric reads used for downstream analyses.

Sample	Input	Filtered	Input passed filter (%)	Denoised	Merged	Input merged (%)	Non-chimeric	Input non-chimeric (%)
***Ca*1**	58194	39989	68.72	35582	22470	38.61	11775	20.23
***Ca*2**	60006	42828	71.37	37994	23497	39.16	13267	22.11
***Tg*1**	54766	40215	73.43	36283	24083	43.97	12502	22.83
***Tg*2**	49245	37954	77.07	34948	25482	51.75	9273	18.83
**Up**	63948	45695	71.46	41563	29779	46.57	15648	24.47
**Total**	**286159**	**206681**		**186370**	**125311**		**62465**	

Table S2

Mean concentration values ± standard deviation of physicochemical parameters for each experimental treatment (*Tg, Ca*, and Unplanted) and influent wastewater.

Sample	Temperature (°C)	pH (Units)	DO (mg · L^{-1})	NH_4^+–N (mg · L^{-1})	PO_4^{3-} (mg · L^{-1})
Influent	26.1 ± 5.1	8.1 ± 0.8	0.58 ± 0.77	54.4 ± 34.7	14.0 ± 8.3
*Ca*1	25.95 ± 5.6	7.45 ± 0.1	4.30 ± 1.3	25.50 ± 20.7	9.40 ± 11.3
*Ca*2	25.95 ± 5.6	7.45 ± 0.1	4.30 ± 1.3	25.50 ± 20.7	9.40 ± 11.3
*Tg*1	26.05 ± 5.2	7.45 ± 0.6	3.30 ± 1.4	15.40 ± 16.7	6.80 ± 11.5
*Tg*2	26.05 ± 5.2	7.45 ± 0.6	3.30 ± 1.4	15.40 ± 16.7	6.80 ± 11.5
Up	25.05 ± 5.7	7.90 ± 0.8	3.80 ± 1.7	32.30 ± 22.4	8.90 ± 5.6

biology

MDPI

Table S3
Topological properties of co-occurrence networks in the root microbiomes of macrophytes from a pilot-scale CW.

ASVs Level Family	Network features					Taxonomy		
	Betweenness centrality	Closeness centrality	Degree	Modularity class	Strongly Connected	Class	Order	Genus/Specie
Rhodocyclaceae	19943.86	0.48	137	1	440	*γ-Proteobacteria*	*Burkholderiales*	*Sulfurisoma*
Chitinophagaceae	15324.44	0.53	228	4	84	*Sphingobacteria*	*Sphingobacteriales*	*Not assigned*
Margulisbacteria	11588.25	0.59	327	0	133	*Not assigned*	*Not assigned*	*Not assigned*
Xanthomonadaceae	11564.29	0.50	269	3	805	*γ-Proteobacteria*	*Xanthomonadales*	*Pseudoxanthomonas*
Rhodocyclaceae	9402.27	0.55	259	3	922	*γ-Proteobacteria*	*Burkholderiales*	*Not assigned*
Rhodocyclaceae	8587.93	0.48	327	0	85	*γ-Proteobacteria*	*Burkholderiales*	*Denitratisoma*
Devosiaceae	8293.94	0.53	262	3	437	*α- Proteobacteria*	*Rhizobiales*	*Devosia*
Comamonadaceae	5597.96	0.47	262	3	818	*γ-Proteobacteria*	*Burkholderiales*	*Not assigned*
Rhodocyclaceae	4667.47	0.47	244	2	728	*γ-Proteobacteria*	*Burkholderiales*	*Sulfurisoma*
NS11-12 marine group	4660.86	0.53	233	4	982	*Bacteroidia*	*Sphingobacteriales*	*NS11-12 marine group*
Gallionellaceae	4631.99	1.00	461	0	10	*γ-Proteobacteria*	*Burkholderiales*	*Sideroxydans*
Planctomycetaceae	4408.52	0.51	225	4	401	*Planctomycetes*	*Pirellulales*	*Pir4 lineage*
Uncultured anaerobic	3816.47	0.50	233	4	804	*Chloroflexia*	*Thermomicrobiales*	*JG30-KF-CM45*
Uncultured	3816.47	0.50	233	4	803	*α- Proteobacteria*	*Uncultured*	*Lacibacterium aquatile*
PHOS-HE36	3760.11	0.67	245	2	680	*Ignavibacteria*	*Ignavibacteriales*	*Not assigned*
Saprospiraceae	3678.50	0.50	244	2	460	*Bacteroidia*	*Chitinophagales*	*Not assigned*
A4b_OLB15	3494.54	0.45	225	4	1052	*Anaerolineae*	*SBR1031*	*Chloroflexi bacterium*
Uncultured bacterium	3172.08	0.51	227	4	75	*Anaerolineae*	*SBR1031*	*A4b-Uncultured*
Uncultured proteobacterium	2915.62	0.47	269	3	983	*γ-Proteobacteria*	*R7C24*	*Uncultured*
Sphingomonadaceae	2888.12	0.34	133	1	477	*α- Proteobacteria*	*Sphingomonadales*	*Novosphingobium*
Christensenellaceae	2610.30	0.33	133	1	474	*Clostridia*	*Christensenellales*	*Uncultured_sludge*

Anaerolineaceae	2142.59	0.55	244	2	391	*Anaerolineae*	*Anaerolineales*	*Bellilinea*
PHOS-HE36	2120.31	0.50	133	1	386	*Ignavibacteria*	*Ignavibacteriales*	*Not assigned*
Chitinivibrionia uncultured	2010.34	0.39	238	2	442	*Chitinivibrionia*	*Uncultured*	*Uncultured*
Rhodocyclaceae	1724.23	1.00	134	1	15	*γ-Proteobacteria*	*Burkholderiales*	*Not assigned*
Env OPS_17	1685.43	0.62	233	4	61	*Bacteroidia*	*Sphingobacteriales*	*Uncultured Alistipes*
Gaiellaceae	1633.96	0.59	233	4	59	*Thermoleophilia*	*Gaiellales*	*Gaiella occulta*
Rhodocyclaceae	1555.97	0.31	133	1	462	*γ-Proteobacteria*	*Burkholderiales*	*Not assigned*
Nitrospiraceae	1336.88	0.55	225	4	1130	*Nitrospiria*	*Nitrospirales*	*Nitrospira japonica*
Pedosphaeraceae	1175.02	0.58	326	0	17	*Verrucomicrobiae*	*Pedosphaerales*	*Uncultured soil*
Clostridiaceae	939.73	0.50	233	4	54	*Clostridia*	*Clostridiales*	*C. sensu stricto*
Alicyclobacillaceae	843.26	0.61	245	2	71	*Bacilli*	*Alicyclobacillales*	*Tumebacillus*
Bacillaceae	843.26	0.63	245	2	72	*Bacilli*	*Bacillales*	*Bacillus*
Blastocatellaceae	686.50	0.61	329	0	11	*Blastocatellia*	*Blastocatellales*	*Stenotrophobacter*
SBR1031 uncultured	602.185	0.66	259	3	39	*Anaerolineae*	*SBR1031*	*Uncultured sludge*
Ferrovibrionaceae	544.80	0.63	269	3	57	*α- Proteobacteria*	*Ferrovibrionales*	*Ferrovibrio*
Alicyclobacillaceae	528.06	0.61	269	3	51	*Bacilli*	*Alicyclobacillales*	*Tumebacillus*
Rhizobiaceae	528.06	0.58	269	3	50	*α- Proteobacteria*	*Rhizobiales*	*Not assigned*
Kineosporiaceae	313.14	0.48	135	1	396	*Actinobacteria*	*Kineosporiales*	*Not assigned*
Rhodobacteraceae	190.79	0.38	135	1	472	*α- Proteobacteria*	*Rhodobacterales*	*Paracoccus*
Xanthobacteraceae	190.79	0.37	135	1	464	*α- Proteobacteria*	*Rhizobiales*	*Not assigned*
HOC36 uncultured	70.15	1.00	245	2	14	*γ-Proteobacteria*	*HOC36*	*Uncultured soil*
Gallionellaceae	70.15	1.00	245	2	13	*γ-Proteobacteria*	*Burkholderiales*	*Sideroxydans*
Pirellulaceae	2.80	0.39	7	3	399	*Planctomycetes*	*Pirellulales*	*Pir4 lineage*
Rhizobiaceae	2.32	0.42	135	1	499	*α- Proteobacteria*	*Rhizobiales*	*Not assigned*
Chitinophagaceae	0.00	0.71	327	0	297	*Bacteroidia*	*Chitinophagales*	*Terrimonas*
Anaerolineaceae	0.00	0.75	327	0	362	*Anaerolineae*	*Anaerolineales*	*Uncultured delta*
Uncultured Acidobacterium	0.00	0.70	327	0	275	*Vicinamibacteria*	*Vicinamibacterales*	*Uncultured Acidobacterium*
Rhizobiales Incertae Sedis	0.00	0.57	127	1	387	*α- Proteobacteria*	*Rhizobiales*	*Uncultured Rhizobiales*
Microscillaceae	0.00	0.35	127	1	517	*Bacteroidia*	*Cytophagales*	*Hassallia byssoidea*
Leptospiraceae	0.00	0.51	135	1	568	*Leptospirae*	*Leptospirales*	*Leptonema*

Sporichthyaceae	0.00	0.46	135	1	530	*Actinobacteria*	*Frankiales*	*Longivirga aurantiaca*
Rhodomicrobiaceae	0.00	0.45	135	1	519	*α- Proteobacteria*	*Rhizobiales*	*Rhodomicrobium*
Myxococcaceae	0.00	0.00	245	2	12	*Myxococcia*	*Myxococcales*	*P3OB-42 uncultured_soil*
Burkholderiales SC-I-84	0.00	0.00	248	2	2	*γ-Proteobacteria*	*Burkholderiales*	*Not assigned*
Beijerinckiaceae	0.00	0.00	269	3	37	*α- Proteobacteria*	*Rhizobiales*	*Microvirga*
Sphingomonadaceae	0.00	0.60	230	4	1191	*α- Proteobacteria*	*Sphingomonadales*	*Altererythrobacter*
Xanthobacteraceae	0.00	0.50	14	4	58	*α- Proteobacteria*	*Rhizobiales*	*uncultured"*

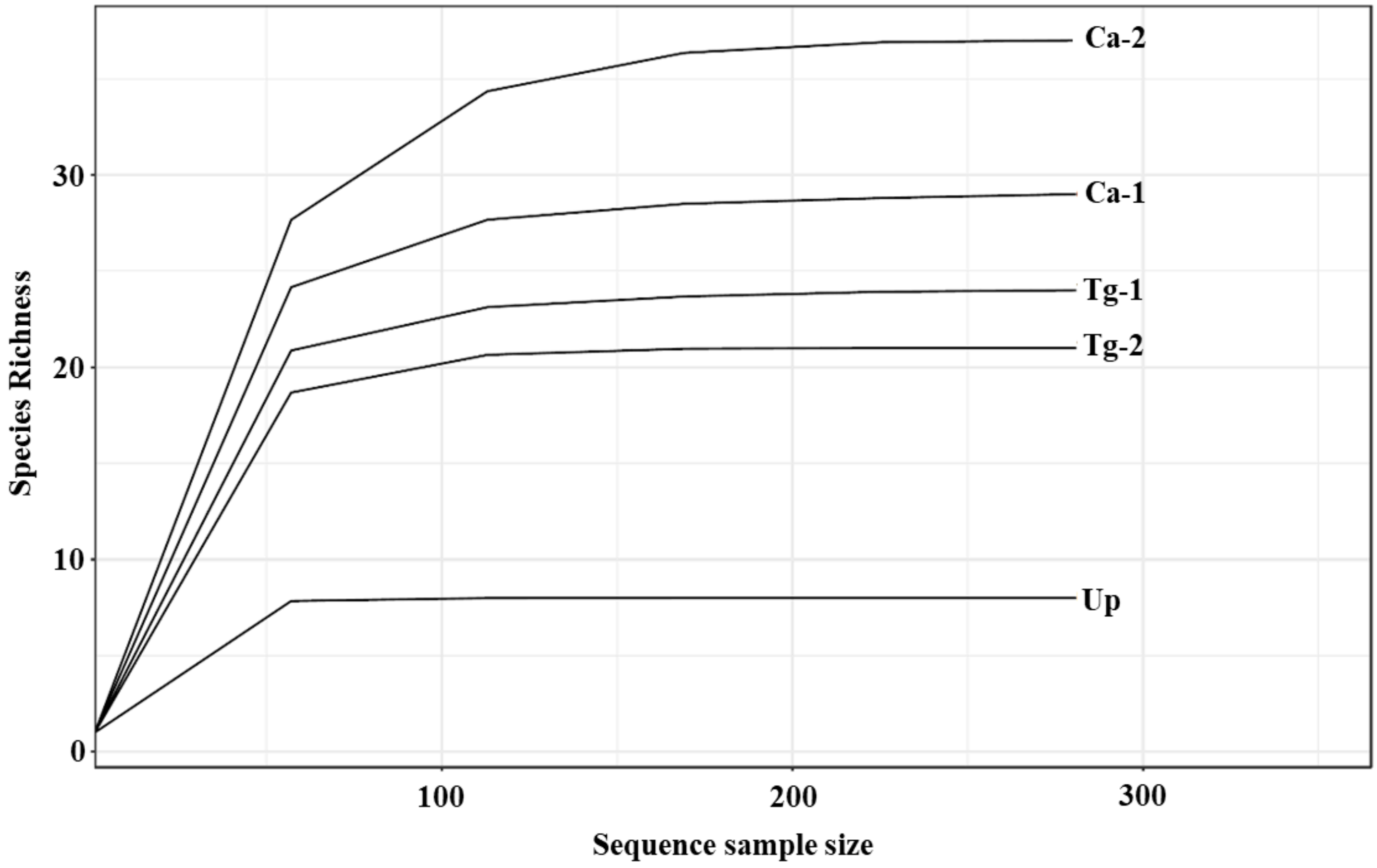

Figure S1. Rarefaction curve showing the bacterial species richness observed from different experimental treatments in a pilot wetland plant.

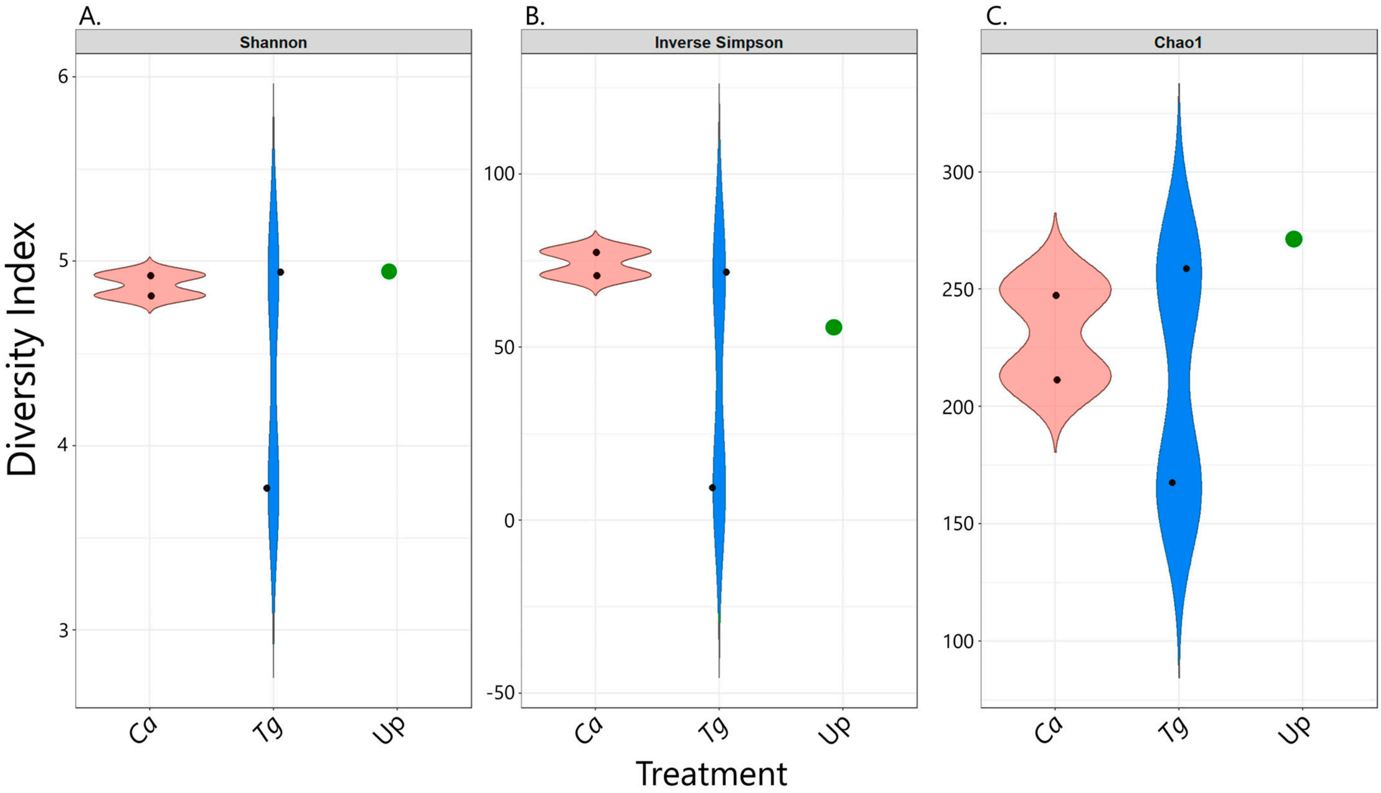

Figure S2. Diversity indices at the family level in the root microbiomes of macrophytes (*C. articulatus* and *T. geniculata*) across different experimental treatments in a pilot wetland study (n=5). The indices include (**A**). Shannon, (**B**). Inverse Simpson, (**C**). Chao1, offering comprehensive insights into the microbial community structure.
